# Alternative Endoscopy Reading Paradigms Determine Score Reliability and Effect Size in Ulcerative Colitis

**DOI:** 10.1093/ecco-jcc/jjad134

**Published:** 2023-08-24

**Authors:** Walter Reinisch, Vivek Pradhan, Saira Ahmad, Zhen Zhang, Jeremy D Gale

**Affiliations:** Department of Internal Medicine III, Division Gastroenterology & Hepatology, Medical University of Vienna, Vienna, Austria; Statistics, Global Biometry and Data Management, Pfizer Inc., 1 Portland St, Cambridge, MA 02139, USA; Statistics and Programming, Quanticate, Hitchin, UK; Statistics, Global Biometry and Data Management, Pfizer Inc., 1 Portland St, Cambridge, MA 02139, USA; Inflammation and Immunology Research Unit, Pfizer Inc., 1 Portland St, Cambridge, MA 02139, USA

## Abstract

**Objective:**

Central reading of endoscopy is advocated by regulatory agencies for clinical trials in ulcerative colitis [UC]. It is uncertain whether the local/site reader should be included in the reading paradigm. We explore whether using locally- and centrally-determined endoscopic Mayo subscores [eMS] provide a reliable final assessment and whether the paradigm used has an impact on effect size.

**Methods:**

eMS data from the TURANDOT [NCT01620255] study were used to retrospectively examine seven different reading paradigms (using the scores of local readers [LR], first central readers [CR1], second central readers [CR2], and various consensus reads [ConCR]) by assessing inter-rater reliabilities and their impact on the key study endpoint, endoscopic improvement.

**Results:**

More than 40% of eMS scores between two trained central readers were discordant. Central readers had wide variability in scorings at baseline (intraclass correlation coefficient [ICC] of 0.475 [0.339, 0.610] for CR1 vs CR2). Centrally-read scores had variable concordance with LR (LR vs CR1 ICC 0.682 [0.575, 0.788], and LR vs CR2 ICC 0.526 [0.399, 0.653]). Reading paradigms with LR and CR which included a consensus, enhanced ICC estimates to >0.8. At Week 12, without the consensus reads, the CR1 vs CR2 ICC estimates were 0.775 [0.710, 0.841], and with consensus reads the ICC estimates were >0.9. Consensus-based approaches were most favourable to detect a treatment difference.

**Conclusion:**

The ICC between the eMS of two trained and experienced central readers is unexpectedly low, which reinforces that currently used central reading processes are still associated with several weaknesses.

## 1. Introduction

In clinical trials of potential new treatments for ulcerative colitis, central reading of endoscopy as mandated by regulatory agencies such as the FDA [the US Food and Drug Administration] or EMA [the European Medicine Agency] is a standard process to enable the objective assessment of luminal disease severity. The local reader’s role has diminished greatly due to suggestions of bias by other authors as well as regulatory agencies.

As an assessment tool, the FDA and EMA endorse the application of either the Mayo endoscopic subscore [eMS] or the Ulcerative Colitis Endoscopic Index of Severity [UCEIS] score, with the former being used predominantly.^1^ The eMS categorises disease severity on a scale from 0 to 3, based on the presence of the features visible on endoscopy, specifically erythema, vascular pattern, friability, erosion, ulcer, and spontaneous bleeding [Appendix 1].^2^

Despite its widespread use, the eMS has weaknesses. It was not developed with prognostic intent, never underwent proper validation, and lacks the range to depict more precisely the spectrum of endoscopic severity. Further, there is the inherent issue of inter-rater variabilities.^2^ Until 2009,^3^ clinical trials involved locally-read results only, but in recent years, use of centrally-read or combinations of centrally- and locally-read scores have been implemented.

Centrally-read endoscopy scores are determined by off-site readers who are independent and blinded to all study details. Typically, the central reading is provided via a third-party vendor having access to a pool of trained readers and an adequate infrastructure in place to allow the collection of endoscopic videos from different sites across the globe, making them available to the readers almost in real-time. In contrast, locally-read scores are conducted by the site endoscopist who is likely to have knowledge of the study details and the patient’s medical history. Thus, results from central reading are expected to be less biased compared with the corresponding locally-read result. Furthermore, it is expected that the results are less discordant as a consequence of the specific training of central readers using reader charters, implying reduced variability of the scoring indices among different central readers. Therefore, central reading vendors claim that they can offer clinical trial sponsors certain levels of reading accuracy or inter-rater correlations (intraclass correlation coefficient [ICC], Appendix 2) of their readings. Having said this, local readers have the advantage of real-time readings, and thus they have the benefit of being able to redo certain aspects of the endoscopy if the visuals of endoscopy reading are suboptimal [maneuvre the camera to ensure a clearer visual, control the speed of the camera, etc.]. If there is no local read conducted, then readers face the challenge of using endoscopy visuals that may be unclear and thus do not facilitate accurate scoring. Issues such as poor washing of the bowels, fast movement of the camera within the colon, and rapid withdrawal of the camera cannot be addressed by a central reader once they receive the video from the site.

The current practice in clinical trials is that more than one reader [either site plus a central reader or a minimum of two central readers] is assigned to determine the endoscopy score in order to achieve greater reliability and remedy potential inter-reader disagreement. Several approaches have been used to arrive at a final endoscopic score when more than one reader is involved in endoscopy analysis, as becomes inevitable in large, multicentre or international trials. These are described in detail in Gottlieb K, *et al*,^2^ but summarised below.

- If both readers independently arrive at the same score:
  - score is taken as final, and no other action is required.
- If both readers do not arrive at the same score, any of the following may be used:
  - consensus-based approach: ‘a panel looks at the image or other matter of interest and comes, after open deliberation, to a conclusion’;
  - adjudication: a third person is asked to act as a ‘judge’ and decide, using the assessments from the initial two readers and taking them into consideration;
  - voting: voting algorithms use two readers and, in cases of disagreement, an optional third reader [2 + 1 reader algorithm] is added^2,4^; the third reader independently votes on what the score should be, without the knowledge that there was a disagreement between Reader 1 and Reader 2, and the third reader’s independent score is taken as final.

For endoscopic evaluation in Crohn’s disease, various reading paradigms have been tested against each other,^5^ whereas for ulcerative colitis, comparative data on various reader paradigms are not available.

The use of one of the scoring paradigms outlined above, namely the consensus-based approach, and the effect it can have on the ICC coefficient measuring the inter-rater variabilities, are explored in this article, using data from a recently conducted clinical trial in ulcerative colitis, TURANDOT.^6^

## 2. Methods

### 2.1. The TURANDOT study

The TURANDOT study is a Phase 2b, randomised, double-blind, placebo-controlled, multicentre study of the anti-MAdCAM-1 monoclonal antibody ontamalimab [PF-00547659], with five treatment arms: placebo, 7.5 mg, 22.5 mg, 75 mg, and 225 mg administered subcutaneously every 4 weeks.^6^ All 357 enrolled patients provided written consent for participation in TURANDOT, were aged 18–65 years, had a ≥3-month history of active ulcerative colitis extending >15 cm beyond the anal verge, with a total Mayo score ≥6 and a Mayo endoscopic subscore ≥2 at baseline, and had failed or were intolerant to ≥1 conventional therapy. The primary endpoint of this study was clinical remission at Week 12 defined by a total Mayo score ≤2 with no individual subscore >1. A key secondary endpoint, and one which is the focus of the present analysis, was mucosal healing defined by Mayo endoscopic score ≤1, nowadays re-classified as endoscopic improvement. The original version of the Mayo endoscopic scoring system was applied in this study [Appendix 1].

All endoscopies performed by the site endoscopist were also assessed under the same criteria as set forth for central reading, with the exception that they were not blinded. The endoscopic videos/images were subsequently uploaded to a central repository, managed by a third-party vendor for the central reading. The central readers were completely blinded to all study details, including whether the endoscopy video was from baseline or Week 12. All endoscopic assessments performed by the site endoscopist are labelled as local read [LR]. All endoscopic assessments performed by remote readers of the third-party are labelled as central read [CR]. A score arrived at via consensus of the central reads is labelled consensus central read [ConCR].

### 2.2. Different sources

For this analysis, the aim was to explore the impact of different sources of endoscopic readings on one of the key secondary endpoints in the TURANDOT study, namely endoscopic improvement. Seven reading paradigms, presented in Table 1 below, were explored.

**Table 1.** Proposed reading paradigms.

1. High-volume reader	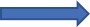	- The central reader who read most of the endoscopy videos - All scores from these readers are labelled as the High-volume reader
2. Local read [LR]	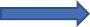	- The score given by the local endoscopist
3. Central read 1 [CR1]	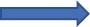	- Score given by a central reader on the first round of readings
4. Central read 2 [CR2]	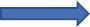	- Score given by a central reader on the second round of readings
5. Consensus central read [ConCR]	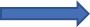	- If scores for CR1 = CR2 → this score is used - If scores for CR1 ≠ CR2 → consensus call held with the original two central readers and an additional reader to determine the final score after open deliberation
6. Consensus LR/CR1 [ConLC1]^a^	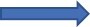	- If scores for LR = CR1 → this score is used - If scores for LR ≠ CR1 → ConCR used - CR2 score used if ConCR missing
7. Consensus LR/CR2 [ConLCR2]^a^	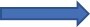	- If scores for LR = CR2 → this score is used - If scores for LR ≠ CR2 → ConCR used - CR1 score used if ConCR missing

^a^It should be noted that ConLCR1 and ConLCR2 use the LR to determine a final score but they are not formal ‘consensus’ results arrived at by a panel of readers as with ConCR.

From the TURANDOT study, the central reader who read most of the endoscopy videos [for this study] was labelled the High-volume reader. LR, CR1, CR2, and ConCR are as defined within Table 1.

The intention was to explore what effect the High-volume reader, LR, CR1, CR2, ConCR, and the final two combinations proposed in the table below, had on the ICC, and if any of the approaches could lead to a robust final score.

**Figure 1.**
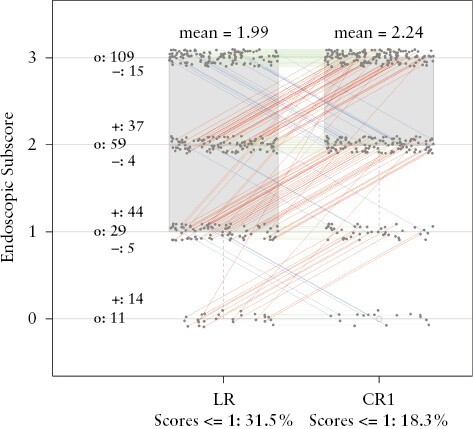
Distribution of local read [LR] and central read 1 [CR1] endoscopic score at Week 12. Red lines show increase in scoring by CR1 compared with LR. Blue lines show decrease in scoring by CR1 compared with LR. Green lines indicate no difference between scoring of LR and CR1. CR1 mostly provided a score higher by 1 compared with the LR score, which resulted in a higher mean score of CR1 [2.24] than LR [1.99]. Less prevalent were instances where CR1 gave a lower score than LR by a maximum of 1. The endoscopic improvement rate based on LR was 31.5%, whereas based on CR1 it was 18.3%. The frequency of LR and CR1 agreeing on endoscopy subscores at Week 12 was 63.6%.

All endoscopic video images uploaded to the central repository were initially assessed by a central reader [referred to as Central Reader 1 or CR1]. Before unblinding the study, in order to ensure the quality of the scoring, some of the centrally-read endoscopy subscores were cross-checked against the locally-read subscores, and wide variability was detected. For example, there were several endoscopic video images at Week 12 where a score of 0 [indicating endoscopic remission] was assigned by the local reader; however, when the same video was scored by the Central Reader [CR1], the highest possible score of 3 [indicating severe disease activity] was assigned [Figure 1]. The readings conducted at baseline also showed large inter-rater variability [Table 1], highlighting the potential disconnects present even at the stage of determining participant eligibility. Such disparity between the scorings of the same endoscopy examination brought into question the reliability of the results. As a consequence of these scoring discrepancies, all videos were re-read by a second set of blinded central readers from the same vendor [referred to as Central Reader 2 or CR2]. There were five central readers who contributed to this study. CR1 and CR2 were selected from this pool of five readers based on their availabilities.

Since all central readers were trained and certified using the same training protocols, there was an expectation of reduced variation between the readers. However, in this study more than 40% of the scores from CR1 and CR2 were observed to be discordant. As a consequence of the scoring inconsistencies between CR1 and CR2, a panel of readers was engaged through video conferencing [before unblinding] to generate a consensus score whenever there was a score difference between CR1 and CR2. The panel consisted of three central readers: the original CR1, CR2, and a third CR. A consensus score was arrived at after open deliberation by CR1, CR2, and the third reader via video call. Therefore, whenever the reading indices [scores] of CR1 and CR2 were in exact agreement, this score was assigned as the final score; if not, the consensus score was used as the final score. The final score obtained in this process is labelled the Consensus Central Read [ConCR].

### 2.3. Study population

All participants in the modified intention to treat [mITT] groups, at baseline and Week 12, were considered for the analyses. The mITT population was defined as those randomised participants who received at least one dose of investigational product.

### 2.4. Statistical analyses

The correlation or the inter-rater reliability between any two from the seven reading paradigms at baseline and at Week 12 were assessed using the ICC and the corresponding 95% confidence intervals. For the estimation of these ICCs, a proportional-odds, cumulative logit model with a subject-specific random effect was fitted to the ordinal response scores using PROC NLMIXED [SAS 9.4, SAS Institute, Cary, NC]. We found that the cumulative logit model, by treating the scores as ordinal response, outperformed the normal model for continuous response with smaller Akaike information criterion [AIC] values [further details provided in Appendix 4]. The estimated ICC based on the model is the proportion of the subject variation in the total variation which further integrates the variation due to the two reading paradigms. Therefore, a higher value for ICC indicates a relatively lower paradigm-specific variability and hence a higher degree of agreement.

Further, based on each of the seven reading paradigms, the key secondary endpoint endoscopic improvement at Week 12, of different dose groups was compared with the placebo group using the exact unconditional method proposed by Chan and Zhang [1999].^7^

## 3. Results

Of the 357 participants enrolled in the TURANDOT study, four participants were excluded from the analysis population [mITT] due to missing locally- or centrally-read endoscopy scores. Hence, a total of 353 participants was selected retrospectively for these analyses at baseline and 327 participants were included at Week 12 in most reading paradigms, with the exception of the High-volume reader [an individual who read 71.7% of all endoscopy videos each at baseline and Week 12] [Table 2].

**Table 2.** Disposition summary at baseline and Week 12.

	Placebo	7.5 mg	22.5 mg	75 mg	225 mg	Total
**Number of readers, *n* [%]**						
Baseline [N]	73	71	72	71	70	357
High-volume reader	55 [75.3]	49 [69.0]	51 [70.8]	47 [66.2]	54 [77.1]	256 [71.7]
LR	73 [100.0]	70 [98.6]	71 [98.6]	70 [98.6]	69 [98.6]	353 [98.9]
CR1	73 [100.0]	70 [98.6]	71 [98.6]	70 [98.6]	69 [98.6]	353 [98.9]
CR2	73 [100.0]	70 [98.6]	71 [98.6]	70 [98.6]	69 [98.6]	353 [98.9]
ConCR	73 [100.0]	70 [98.6]	71 [98.6]	70 [98.6]	69 [98.6]	353 [98.9]
ConLCR1	73 [100.0]	70 [98.6]	71 [98.6]	70 [98.6]	69 [98.6]	353 [98.9]
ConLCR2	73 [100.0]	70 [98.6]	71 [98.6]	70 [98.6]	69 [98.6]	353 [98.9]
Week 12 [N]	67	63	69	67	63	329
High-volume reader	51 [76.1]	42 [66.7]	49 [71.0]	44 [65.7]	50 [79.4]	236 [71.7]
LR	67 [100.0]	62 [98.4]	69 [100.0]	66 [98.5]	63 [100.0]	327 [99.4]
CR1	67 [100.0]	62 [98.4]	69 [100.0]	66 [98.5]	63 [100.0]	327 [99.4]
CR2	67 [100.0]	61 [96.8]	68 [98.6]	66 [98.5]	63 [100.0]	325 [98.8]
ConCR	67 [100.0]	62 [98.4]	69 [100.0]	66 [98.5]	63 [100.0]	327 [99.4]
ConLCR1	67 [100.0]	62 [98.4]	69 [100.0]	66 [98.5]	63 [100.0]	327 [99.4]
ConLCR2	67 [100.0]	62 [98.4]	69 [100.0]	66 [98.5]	63 [100.0]	327 [99.4]

Percentages are based on N.

LR, local read; CR1, central read 1; CR2, central read 2; ConCR, consensus central read; ConLCR1, score determined using primarily LR, CR1, and ConCR; ConLCR2, score determined using primarily LR, CR2, and ConCR; mITT, modified intention to treat; N, number of mITT participants in the Clinical Study Report with non-missing observed subscore at specified visit; N [%], number of mITT participants in this analysis with non-missing observed subscore for each reading method at specified visit.

**Figure 2.**
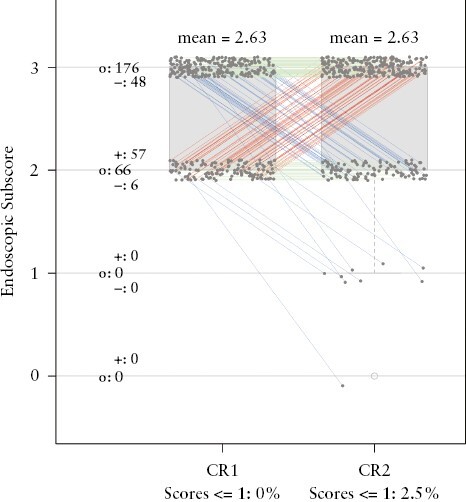
Distribution of central read 1 [CR1] and central read 2 [CR2] endoscopic score at baseline. Red lines show increase in scoring by CR2 compared with CR1. Blue lines show decrease in scoring by CR2 compared with CR1. Green lines indicate no difference between scoring of CR1 and CR2. The frequency of CR1 and CR2 agreeing on endoscopy subscores at baseline was 68.6%. The intraclass correlation coefficient [ICC] for CR1 vs CR2 at baseline was 0.475 [0.339, 0.610], which is considered poor.

**Figure 3.**
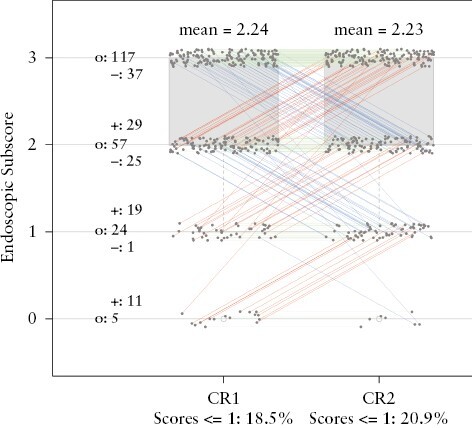
Distribution of central read 1 [CR1] and central read 2 [CR2] endoscopic scores at Week 12. Red lines show increase in scoring by CR2 compared with CR1. Blue lines show decrease in scoring by CR2 compared with CR1. Green lines indicate no difference between scoring of CR1 and CR2.

**Figure 4.**
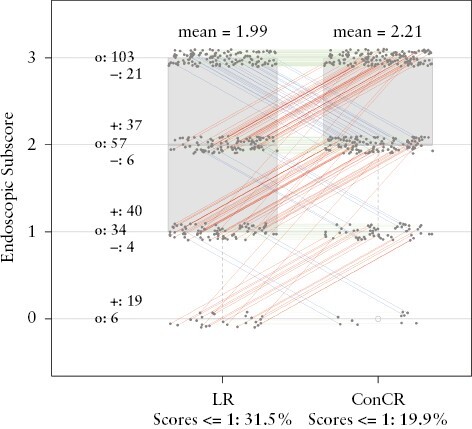
Distribution of local read [LR] and consensus central read [ConCR] scores at Week 12. Red lines show increase in scoring by ConCR compared with LR. Blue lines show decrease in scoring by ConCR compared with LR. Green lines indicate no difference between scoring of LR and ConCR.

Figures 1–4 show the pair-wise distribution of LR, CR1, CR2, and ConCR scores from the TURANDOT study for baseline and the Week 12 time point. Further plots for comparisons at baseline and Week 12 are included in Appendix 3. Each figure comprises two boxplots showing the distribution of scores from two readers, and a superimposed spaghetti plot.

The CR1 vs CR2 figure at baseline [Figure 2 below] highlights one of the consequences of having low inter-rater correlation. It can be seen that nine individuals who were given a Mayo endoscopic subscore of ≥2 by CR1, were subsequently given a score of <2 by CR2. Since having a Mayo endoscopic subscore ≥2 was a key inclusion criterion, this highlights the fact that potentially nine such individuals were included in the TURANDOT study who did not in fact meet the inclusion criteria. It should also be noted that it is due to this inclusion criterion that the baseline plots are densely populated between endoscopic subscores 2 and 3. In comparison, the Week 12 plots, due to improvement in endoscopic disease activity, have scoring spanning across subscores 0 to 3.

Further figures of comparisons at baseline [LR score against CR1, LR score against CR2, and LR score against the ConCR] are included in Appendix 3. Figure 3 shows the distribution of the endoscopic scorings from CR1 and CR2. Even though the mean scores in Figure 3 were similar [2.24 versus 2.23] and the ICC and 95% confidence interval [CI] were better than baseline (0.775 [0.710, 0.841]), there were several study participants for whom quite different scores were given by the two central readers. These differences are apparent in Figure 3. The frequency of CR1 and CR2 agreeing on endoscopy subscores at Week 12 was 62.5%, and thus was actually worse than that at baseline [68.6%].

The final scores were obtained using the ConCR method, and the distribution of LR vs ConCR scores is given in Figure 4. Due to the described disparities between a local read and a central read, the distribution of LR and ConCR scores resembles the relationship between LR and CR1 scores as displayed in Figure 1, showing that even after obtaining a consensus score for the central readings, the scoring taking place at the site is still quite discordant to the central reading score. Overall, the mean score given by ConCR is higher than LR, thus reflecting that a score obtained using a consensus-based paradigm is more conservative and less likely to indicate improvement or absence in disease activity. Thus, Figures 1–4 [and Supplementary Figures 1 to 4 in Appendix 3] demonstrate that there are clear disparities in the determination of endoscopy scores. Currently, there is scant literature available presenting preferred or optimal reading paradigms, which was the motivation behind the analysis presented here.

Table 3 details the ICCs from all seven reading paradigms for endoscopy subscores at baseline and at Week 12. In general, the inter-rater reliability of the endoscopy subscore is lower at baseline versus Week 12, even though overall results are similar. Of note, at baseline, CR1 vs CR2 had the lowest ICC [95% CI], 0.475 [0.339, 0.610], followed by LR vs CR2, 0.526 [0.399, 0.653], and LR vs High-volume reader, 0.577 [0.443, 0.711]. Overall, a single High-volume reader did not perform better, but no worse, than a group of readers from CR1 or CR2. These results reaffirm the scoring disparity within the central readers which is being referred to throughout this paper.

**Table 3:** Inter-rater reliability of endoscopy subscore at baseline and Week 12 [mITT, observed cases].

	Baseline	Week 12
LR vs High-volume reader	0.577 [0.443, 0.711]	0.713 [0.624, 0.802]
LR vs CR1	0.682 [0.575, 0.788]	0.803 [0.746, 0.860]
LR vs CR2	0.526 [0.399, 0.653]	0.701 [0.625, 0.778]
LR vs ConCR	0.694 [0.589, 0.798]	0.791 [0.732, 0.851]
LR vs ConLCR1	0.868 [0.804, 0.932]	0.866 [0.823, 0.908]
LR vs ConLCR2	0.776 [0.689, 0.863]	0.833 [0.783, 0.884]
High-volume reader vs CR1	0.809 [0.724, 0.894]	0.877 [0.827, 0.926]
High-volume reader vs CR2	0.843 [0.768, 0.918]	0.904 [0.863, 0.946]
High-volume reader vs ConCR	0.845 [0.774, 0.917]	0.938 [0.905, 0.972]
High-volume reader vs ConLCR1	0.796 [0.709, 0.884]	0.903 [0.862, 0.945]
High-volume reader vs ConLCR2	0.846 [0.774, 0.917]	0.918 [0.879, 0.958]
CR1 vs CR2	0.475 [0.339, 0.610]	0.775 [0.710, 0.841]
CR1 vs ConCR	0.812 [0.735, 0.888]	0.939 [0.913, 0.965]
CR1 vs ConLCR1	0.937 [0.906, 0.967]	0.976 [0.966, 0.986]
CR1 vs ConLCR2	0.733 [0.636, 0.829]	0.902 [0.866, 0.939]
CR2 vs ConCR	0.881 [0.825, 0.937]	0.936 [0.909, 0.963]
CR2 vs ConLCR1	0.692 [0.592, 0.793]	0.865 [0.820, 0.910]
CR2 vs ConLCR2	0.930 [0.896, 0.965]	0.968 [0.951, 0.984]
ConCR vs ConLCR1	0.952 [0.931, 0.973]	0.978 [0.970, 0.987]
ConCR vs ConLCR2	0.972 [0.959, 0.985]	0.988 [0.980, 0.995]
ConLCR1 vs ConLCR2	0.915 [0.865, 0.965]	0.964 [0.945, 0.982]

mITT represents all subjects randomiesd and who received at least one dose of investigational product, and have both locally- and centrally-read endoscopy scores [four subjects were excluded from the protocol-defined mITT due to missing locally- or centrally-read endoscopy score].

Baseline is defined as the latest measurement before first dosing.

The 95% confidence interval of risk difference from placebo is calculated using the method of Chan and Zhang.^7^

LR, local read; CR1, central read 1; CR2, central read 2; ConCR, consensus central read; ConLCR1, score determined using primarily LR, CR1, and ConCR; ConLCR2, score determined using primarily LR, CR2, and ConCR; mITT, modified intention to treat.

The highest ICC was for ConCR vs ConLCR2 with an ICC estimate [95% CI] of 0.972 [0.959, 0.985] at baseline and of 0.988 [0.980, 0.995] at Week 12. The second highest correlation was between ConCR scores vs ConLCR1 scores. However, these are to be expected as ConLCR1 and ConLCR2 are based on a consensus in which ConCR is a contributing factor.

The main comparisons of interest are of ConCR with LR, CR1, and CR2. The ICCs [95% CIs] for CR1 vs ConCR, and for CR2 vs ConCR, are substantially improved to 0.812 [0.735, 0.888], and 0.881 [0.825, 0.937], respectively, from the CR1 vs CR2 ICC of 0.475 [0.339, 0.610], showing the benefit of including a consensus score in the determination of a final endoscopic subscore. However, it is interesting to note that the ICC for LR vs ConCR is still relatively low at 0.694 [0.589, 0.789]. This points to the inconsistencies between local and central reading, highlighting that this area is in need of further exploration not covered in this paper.

### 3.1. Impact of endoscopy reading paradigms on determining effect size

Consensus-based paradigms were most favourable to detect treatment differences between the ontamalimab 22.5-mg and 75-mg doses versus placebo.

**Figure 5.**
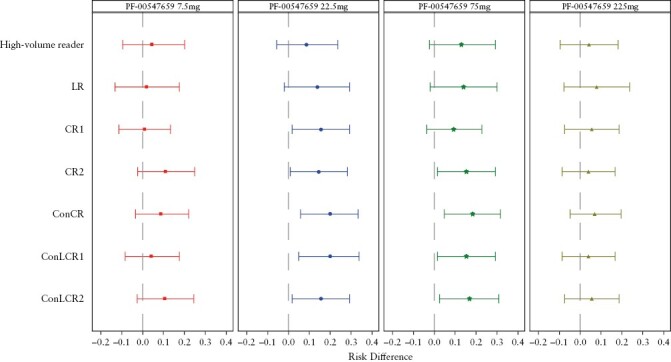
Forest plot of risk difference from placebo in proportion of subjects with endoscopic improvement defined as absolute endoscopic subscore of <=1 point at Week 12 [mITT, observed cases]. mITT represents all subjects randomized and who have received at least one dose of investigational product, and have both local and centrally read endoscopy scores [four subjects were excluded from the protocol defined mITT due to missing local or centrally read endoscopy score]. Baseline is defined as the latest measurement before first dosing. The 95% confidence interval [CI] of risk difference from placebo is calculated using the Chan and Zhang method.^7^ LR, local read; CR1, central read 1; CR2, central read 2; ConCR, consensus central read; ConLCR1, score determined using primarily LR, CR1, and ConCR; ConLCR2, score determined using primarily LR, CR2, and ConCR; mITT, modified intention to treat.

The effects of the paradigms presented in Table 1 on detecting a difference in endoscopic improvement for each treatment arm versus the placebo arm are shown in Figure 5. The 22.5-mg and 75-mg doses were the most efficacious, with the reading paradigms ConCR and ConLCR1 being most preferred for detecting the risk difference as compared with the other paradigms.

These results show that the reading paradigm used does have an impact on the ability to detect an effect size. This is an interesting finding and should be used as a springboard to investigate endoscopy reading paradigms further.

## 4. Discussion

Our results strongly suggest that current applications of central reading in ulcerative colitis clinical trials are unlikely to provide reliable or reproducible results. These findings serve as a clarion call to further develop and standardise endoscopy scoring procedures, and even to review the use of the eMS itself.

For the eMS, some areas for consideration are: the requirement for more rigorous training for the central endoscopy readers; optimising camera and video quality for the endoscopy; further standardization of the entire endoscopy process; and other contributing factors which are discussed in more detail in the paper by Gottlieb *et al.*^2^

One such area was explored in this paper, specifically the aggregation of endoscopy readings from different sources [locally- and centrally-read scores] to generate a final result. Assessing the different methods of aggregation [eg, consensus vs adjudication vs voting] was beyond the scope of this paper; a choice was made to use the consensus-based approach. The proposition was that a single central read was not a reliable enough result, and some sort of influence should be gained from locally-read results as well as further centrally-read results. Increasing numbers of clinical trials rely on central reading only, and therefore are not collecting the local read. However, our data showed that CR1 and CR2 results are often inconsistent, and using reading paradigms that include a consensus of the central reads and some influence from the local read may reduce the variability within the score. At baseline there was such variability in the readings between LR, CR1, and CR2, that some participants were potentially included in the study when they may not have met the inclusion criteria, and similarly some participants may have been excluded when they could possibly have met the inclusion criteria. In contrast, results involving a consensus of central readings consistently provided the best inter-rater reliability both at baseline and at the time point for determination of efficacy, and, in addition, ascribed an optimised effect size for efficacious doses.

The present analysis has limitations. The assessment of the various reader paradigms was post hoc. Also, this was a single trial which involved a single central reading institution; thus, the generalisability of the results should be assessed. Furthermore, TURANDOT was among the first clinical trials applying central reading, and since that time some improvements in reader training and alignment are likely to have been made. However, as standardised procedures have still not been established, a lot of the contemporary trials have not adapted and thus many of the issues are likely to persist. Hence, despite the aforementioned limitations, using the results from 353 and 327 patients at baseline and Week 12, respectively, provides our findings with a meaningful robustness.

We cannot ignore the need for standardisation of the primary aspect of endoscopy reading, namely training for all endoscopy readers for scoring and recording, since ‘the site endoscopist controls the quality of the video recording and lays the foundation for accurate reading for any subsequent evaluations. Better videos may be obtained by asking the site reader to record a video and score it with the knowledge that this will be confirmed or challenged by off-site readers’, as described by Gottlieb *et al.* [2015].^8^ It is time for this approach to be standardised to aid high-quality and robust endoscopic scoring. With the emergence of artificial intelligence in gastrointestinal endoscopy, including scoring of UC endoscopic severity,^9^ our results are also emphasising the necessity to inform those systems on a broad consensus scoring approach, in order to mitigate bias. Our findings also highlight the need for the establishment of alternative UC endoscopic severity scoring systems that are less sensitive to inter-rater disagreement and therefore inconsistencies in the interpretation of a patient’s disease status and its inferred ramifications.

With the FDA maintaining the recommendation for the inclusion of endoscopic evaluation as both an eligibility criterion and a co-primary endpoint for UC clinical trials [and for inflammatory bowel disease more generally], the impact of inaccurate determinations, either to overstate effectiveness, understate it, or to include inappropriate participants, must be addressed. As a consequence, it is imperative that consistent and clear guidelines and procedures are in place to enable reproducible and accurate results.

## 5. Further work

Even though our study is the first describing the performance and impact of various reader paradigms in the assessment of disease activity in an ulcerative colitis clinical trial, this topic shows potential for further exploration. One such area highlighted within this paper is the potential of conducting a formal consensus [using a panel of readers] of the LR and CR results. Although the reading paradigms within this paper were able to reduce variability within the centrally-read results, Table 3 shows that the LR result is still discordant to the overall centrally-read results. This emphasises the potential impact of an LR result and the need to give weight to a site endoscopist when obtaining a final endoscopic result.

Furthermore, it would be interesting to conduct a similar assessment of another scoring system, namely the UCEIS, which is in fact fully validated, using the same study data and video material. UCEIS was not used in the TURANDOT study protocol, but it is claimed to be a better scoring method compared with the eMS, so assessing the inter-rater correlations using the UCEIS would be an important and useful study for the IBD community.

## Supplementary Material

jjad134_suppl_Supplementary_Figure_S1

jjad134_suppl_Supplementary_Figure_S2

jjad134_suppl_Supplementary_Figure_S3

jjad134_suppl_Supplementary_Figure_S4

## Data-Sharing Statement:

Upon request, and subject to review, Pfizer will provide the data that support the findings of this study. Subject to certain criteria, conditions and exceptions, Pfizer may also provide access to the related, individual, de-identified participant data; see [https://www.pfizer.com/science/clinical-trials/trial-data-and-results] for more information.

## Appendix 1

**Endoscopic Mayo Scores:**

0 = normal mucosa or inactive disease

1 = mild disease [erythema, decreased vascular pattern, mild friability]

2 = moderate disease activity [marked erythema, lack of vascular pattern, friability, erosions]

3 = severe disease activity [spontaneous bleeding, ulcerations]

## Appendix 2

To quantify the concordance between the different paradigms, the intraclass correlation coefficient [ICC] calculated was interpreted according to the following categories from Landis and Koch^10^:

**Correlation range interpretation**

<0.00 = poor
0.00–0.2 = slight
0.21–0.4 = fair
0.41–0.6 = moderate
0.61–0.8 = substantial
0.81–1.00 = almost perfect

## Appendix 3

Supplementary Figure 1: Distribution of local read [LR] and central read 1 [CR1] scores at baseline. Red lines show an increase in scoring by CR1 compared with LR. Blue lines show a decrease in scoring by CR1 compared with LR. Green lines indicate no difference between the scoring of LR and CR1.

Supplementary Figure 2: Distribution of local read [LR] and central read 2 [CR2] scores at baseline. Red lines show an increase in scoring by CR2 compared with LR. Blue lines show a decrease in scoring by CR2 compared with LR. Green lines indicate no difference between the scoring of LR and CR2.

Supplementary Figure 3: Distribution of local read [LR] and consensus central read [ConCR] scores at baseline. Red lines show an increase in scoring by ConCR compared with LR. Blue lines show a decrease in scoring by ConCR compared with LR. Green lines indicate no difference between the scoring of LR and ConCR.

Supplementary Figure 4: Distribution of local read [LR] and central read 2 [CR2] scores at Week 12. Red lines show an increase in scoring by CR2 compared with LR. Blue lines show a decrease in scoring by CR2 compared with LR. Green lines indicate no difference between the scoring of LR and CR2.

## Appendix 4

The proportional-odds cumulative logit model we adopted for intraclass correlation coefficient [ICC] estimation is stated as: $logit\left(P\left(Y_{ij}\leq c\right)\right)=\alpha_{c}-\overset{\;p}{\underset{k=1}{\sum }}X_{ijk}\beta_{k}-b_{i}$ for the jth reading paradigm [j=1, 2] for the subject i with score c=0, 1, 2. The random effect due to subject i  is assumed normal $b_{i}∼N\left(0,\sigma^{2}\right)$. The model parameters include the intercept $\alpha_{c}$ for each score c=0, 1, 2 and the slope $\beta_{k}$, k=1 to p  for the p covariates $X_{ijk}\;\;\;$ if any covariate adjustment is considered. Hence ICC$=\sigma^{2}/\left(\sigma^{2}+\pi^{2}/3\right)$ where $\pi^{2}/3\;$ is the variance of the standard logistic distribution under the cumulative logit model.
