# Supplementary figures and images for: Alternative Endoscopy Reading Paradigms Determine Score Reliability and Effect Size in Ulcerative Colitis
Source: J Crohns Colitis. 2023 Aug 24;18(1):82–90. doi: 10.1093/ecco-jcc/jjad134 (PMC10821708; doi:10.1093/ecco-jcc/jjad134)

Endoscopic Subscore

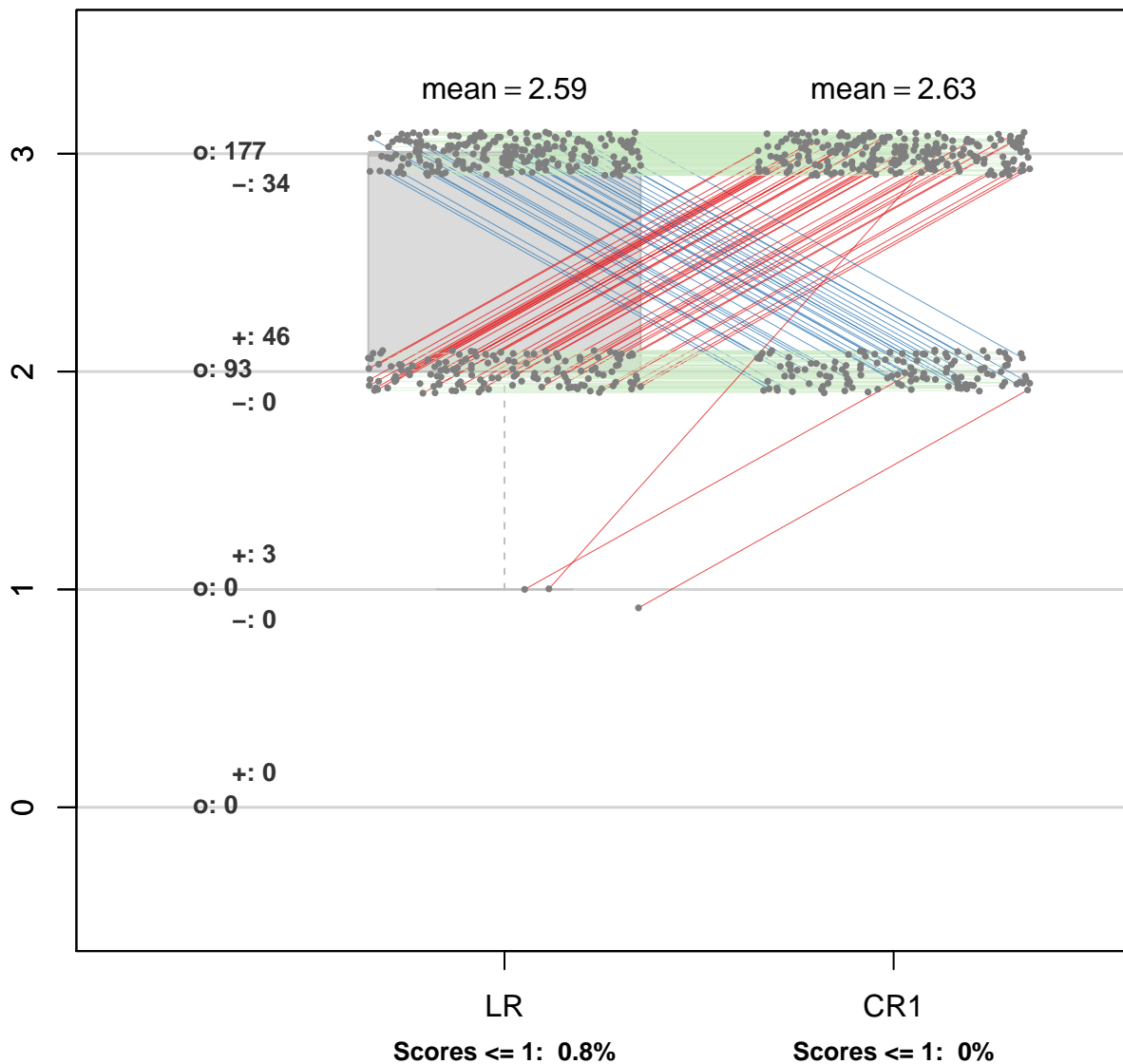

Supplement: jjad134_suppl_Supplementary_Figure_S1 [file jjad134_suppl_supplementary_figure_s1.pdf]

Endoscopic Subscore

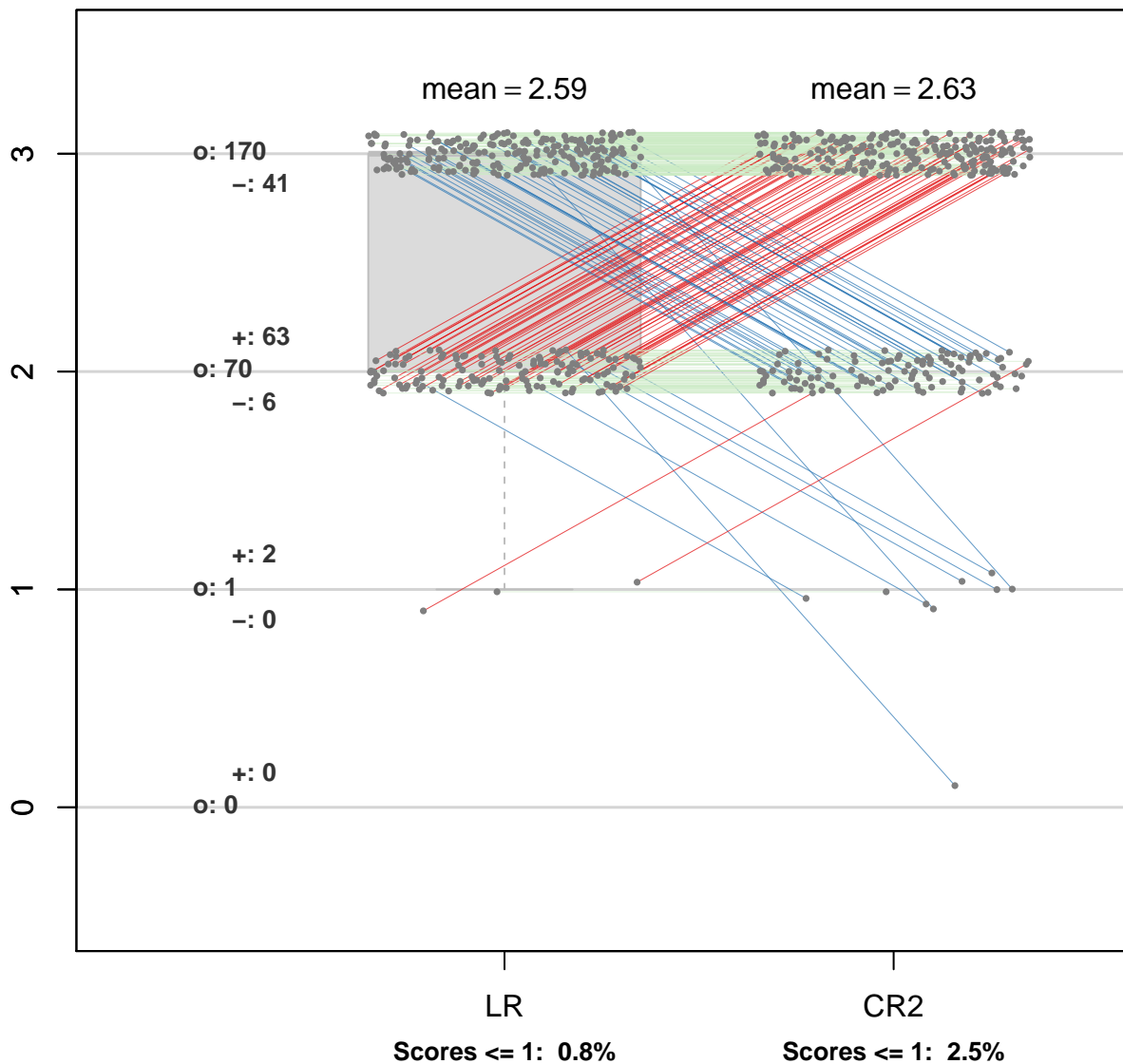

Supplement: jjad134_suppl_Supplementary_Figure_S2 [file jjad134_suppl_supplementary_figure_s2.pdf]

Endoscopic Subscore

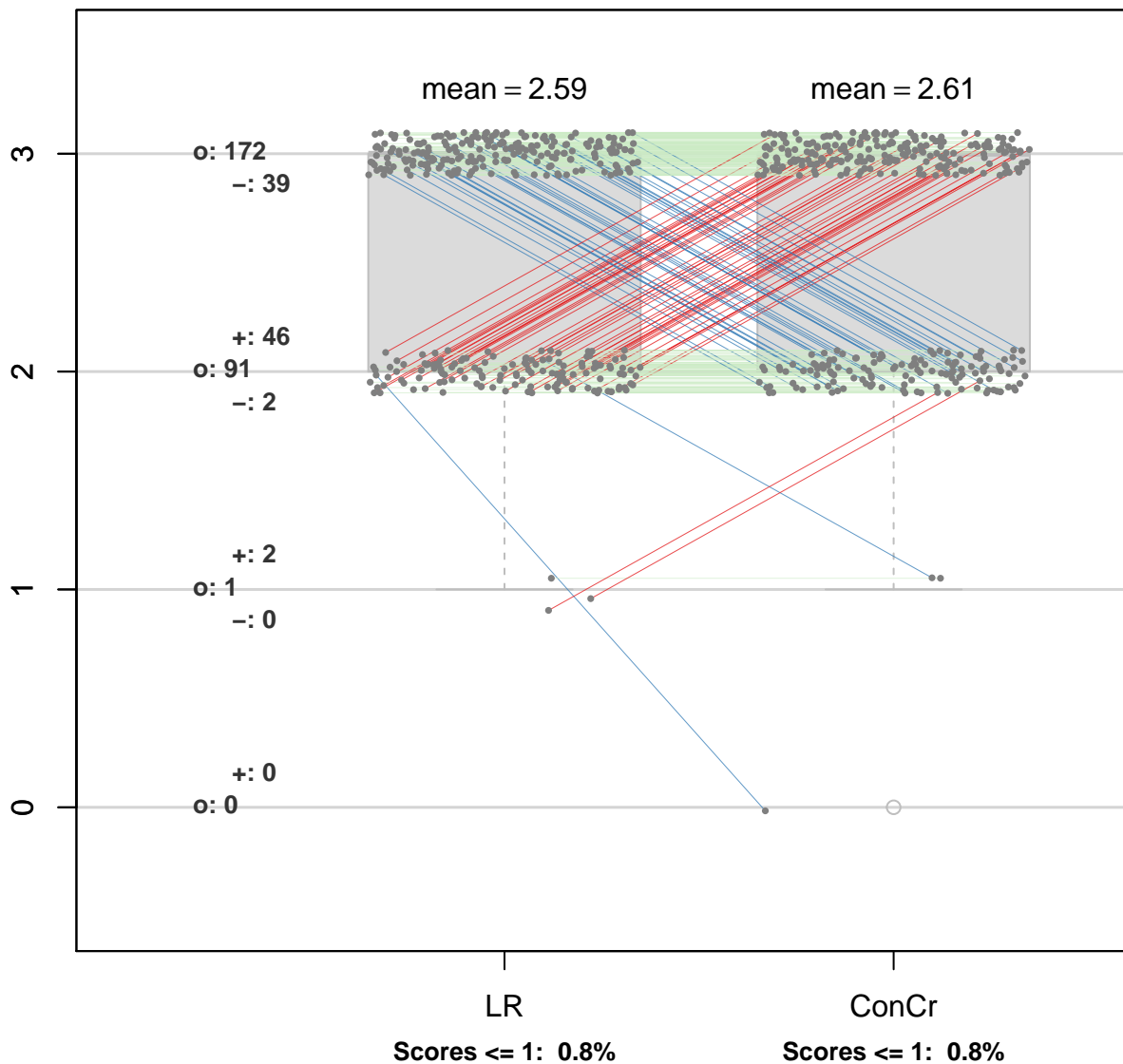

Supplement: jjad134_suppl_Supplementary_Figure_S3 [file jjad134_suppl_supplementary_figure_s3.pdf]

Endoscopic Subscore

3  
2  
1  
0

mean = 1.98

mean = 2.23

o: 99  
-: 24

+: 41  
o: 49  
-: 9

+: 39  
o: 36  
-: 3

+: 21  
o: 4

LR

CR2

Scores <= 1: 31.7%

Scores <= 1: 20.9%

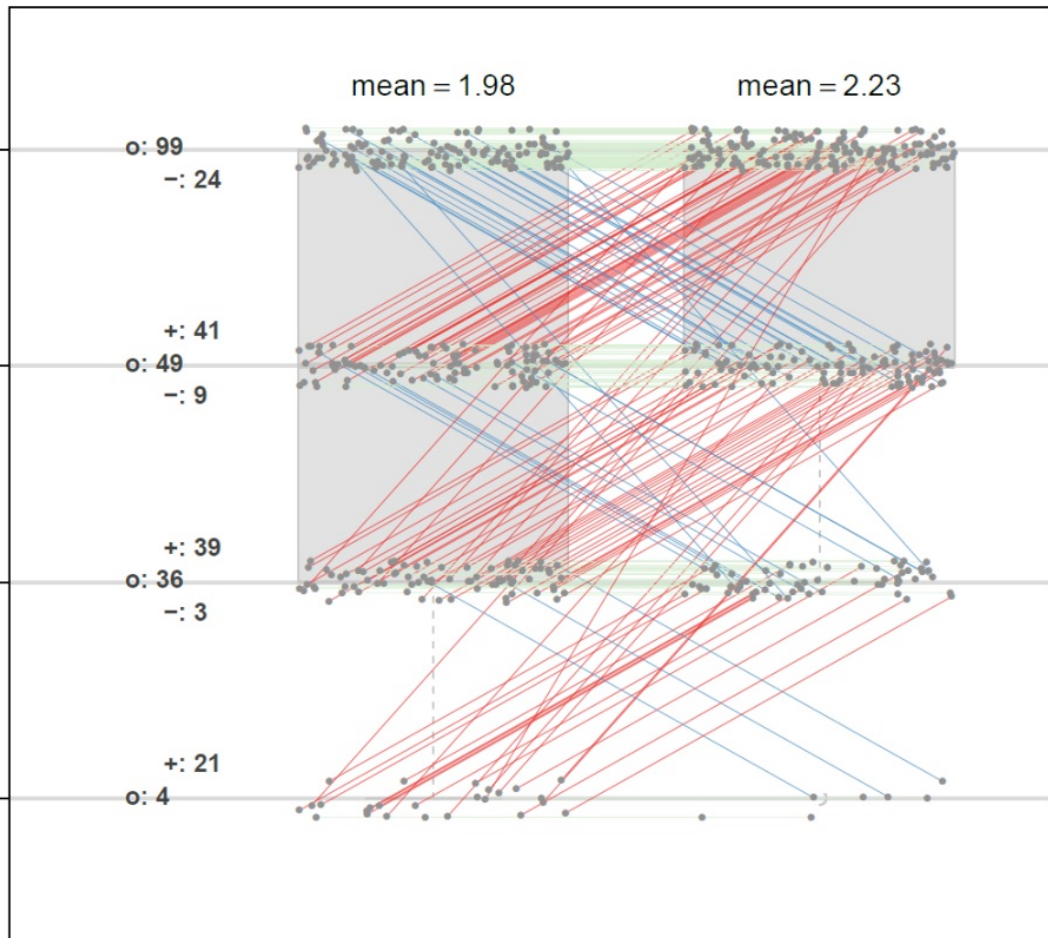

Supplement: jjad134_suppl_Supplementary_Figure_S4 [file jjad134_suppl_supplementary_figure_s4.pdf]
